# A-trait and risk-taking behavior in predicting injury severity among martial arts athletes

**DOI:** 10.3389/fpsyg.2023.1134829

**Published:** 2023-06-30

**Authors:** Ionuț Patenteu, Radu Predoiu, Ryszard Makarowski, Alexandra Predoiu, Andrzej Piotrowski, Adina Geambașu, Sarah Adriana Nica

**Affiliations:** ^1^Faculty of Medicine, University of Medicine and Pharmacy “Carol Davila”, Bucharest, Romania; ^2^Faculty of Physical Education and Sport, Teachers’ Training Department, National University of Physical Education and Sports, Bucharest, Romania; ^3^Faculty of Administration and Social Sciences, Academy of Applied Medical and Social Sciences in Elblag, Elblag, Poland; ^4^Faculty of Physical Education and Sport, Sports and Motor Performance Department, National University of Physical Education and Sports, Bucharest, Romania; ^5^Institute of Psychology, University of Gdańsk, Gdańsk, Poland; ^6^Faculty of Physiotherapy, Special Motricity and Medical Recovery Department, National University of Physical Education and Sports, Bucharest, Romania

**Keywords:** trait anxiety, sports injury, martial arts, risk-taking behavior, athletes

## Abstract

**Introduction:**

Trait anxiety (A-trait) can be seen as a multiplicative function of the person–situation interaction. Considering risk-taking behavior (R-TB), literature highlights instrumental and stimulating risk. The aim of the research is to investigate the level of A-trait (in physically dangerous conditions and in new, unusual situations) and the level of R-TB (instrumental and stimulating risk) in athletes, and to verify to what extent A-trait and risk-taking behavior predict injury severity.

**Materials and methods:**

One hundred and fifty-four senior martial arts athletes from grappling combat sports, striking combat sports and mixed martial artists (MMA) participated in the study. For assessing trait anxiety and risk-taking behavior, the Romanian adaptation of the Endler Multidimensional Anxiety Scales (EMAS), respectively the Romanian adaptation of the Makarowski’s Stimulating and Instrumental Risk Questionnaire were used.

**Results:**

Using multivariate analysis of variance, significant differences between athletes (according to the sports disciplines practiced), in terms of A-trait and R-TB, were examined. Next, we checked the existing correlations between injury severity, A-trait and R-TB scores in athletes practicing striking combat sports, grappling combat sports and MMA. To verify whether there are significant differences in terms of trait anxiety, stimulating and instrumental risk between athletes who have suffered mild, moderate and/or severe injuries and athletes who have suffered only minor/mild injuries, t-Test for Independent Samples was used. Binomial logistic regression procedures were, also, performed, predicting athletes’ likelihood of injury, based on R-TB and A-trait.

**Conclusion:**

A moderate or slightly below average level of anxiety in new, unusual situations and a higher level of instrumental risk are linked with a decreased likelihood of severe injuries in athletes. Martial arts athletes (in entire sample) who have suffered more severe injuries are more adrenaline-seeking in competition and use less rational thinking, taking more pleasure in just performing technical executions, regardless of the outcome.

## 1. Introduction

Combat sports and martial arts are part of the heuristic sport disciplines, requiring for athletes to be inventive, to make fast decisions and carry out appropriate technical-tactical actions in competitions and training, showing cold blood under stressful circumstances (Predoiu et al., 2018). When talking about the relationship between combat sport and martial arts, Kalina (2000) highlighted - “every combat sport is martial arts but not vice versa” (p. 18). With respect to the forms of direct confrontation, martial arts can be divided into two categories (see Predoiu et al., 2022a): Grappling combat sports (judo, jiu-jitsu, freestyle wrestling) which use techniques of throws, ground locks, grips of immobilization of opponent’s body and finishing with joint forcing or choking techniques (Andreato et al., 2017; Coswig et al., 2018) and Striking combat sports (e.g., boxing, kick-boxing, karate, Muay Thai, kenpō, taekwondo, fencing - working with weapons in this case). Considering mixed martial arts (MMA), represents a category which combines techniques from both fighting styles - striking and grappling (Di Bacco et al., 2020).

Combat sports and martial arts have the potential to convey moral values, one of the key elements of martial arts training being the social, ethical and moral development of people practicing (Kostorz and Sas-Nowosielski, 2021).

During a sporting career, competitive athletes may face injuries, a severe injury (or even moderate) being a traumatic event, affecting the psychophysical health in athletes (Brewer, 2007). Injury is seen as “any sports-related musculoskeletal complaint that resulted in an athlete to stop, limit, or modify participation for one or more days” (Li et al., 2015). Also, James and Pieter (2003) defined injury as any situation for which medical assistance was sought, including time-loss injury for a minimum of 1 day. With the increase in the level of performance in sport, an increase in injuries of all kinds was observed (Macedo-Filho et al., 2019; Moriarty et al., 2019). However, in martial arts, as expertise increased the number of injuries decreased (Birrer and Birrer, 1982), a higher frequency of injuries being observed during lower level competitions (when comparing with higher level ones) - Frey et al. (2004).

Injuries in contact sports have a different typology and anatomical regions are affected differently depending on the fighting style (Bledsoe et al., 2006; Del Vecchio et al., 2018). In striking sports injuries are due to limb strikes (fist, elbow, knee, leg), the most common being contusions and tegumentary injuries, with limbs and head (face area) being affected (Hammami et al., 2018; Goes et al., 2020). After a systematic review considering injuries in karate, Thomas and Ornstein (2018) discuss about the following rates/1000AE, in males: lacerations/ contusions/ bruises/ abrasions/ tooth avulsion - 68.1; sprains/ strains - 3.5; epistaxis/ bleeding/ hematomas - 11.4; dislocations - 2.9; concussions - 2.5; fractures - 2.9. The authors assert that “rates of injury/1000AE and/1000 minutesAE were similar for males (111.4/1000AE, 75.4/1000 minAE) and females (105.8/1000AE, 72.8/1000 minAE),” in competitions. Considering boxing (also, a striking combat sport), traumatic brain injuries during competitions are the most severe ones (Jayarao et al., 2010).

In grappling sports the mechanisms are joint sprains and strains, and the typology includes dislocations/subluxations, sprains, ligament injuries, tendon injuries and fractures. The areas affected are the joints of the upper and lower limbs, and intra- and periarticular structures (Pappas, 2007; Jensen et al., 2017; Johnson et al., 2019). In grappling combat sports (e.g., judo), anterior cruciate ligament ruptures are one of the most severe injuries (Lambert et al., 2022). After analyzing the injury incidence during a World Judo Championship, Miarka et al. (2018) found miscellaneous traumas (42.6%) and a higher prevalence of soft-tissue injuries (58.8%) which occurred during gripping fight or when being thrown. A lower prevalence of injuries was observed in the Final, when comparing to Eliminatory phases of the competition. In 2014, researchers (Pocecco et al., 2013) highlighted (following a systematic literature review) that a few retrospective and prospective studies dealing with judo injuries are available in the literature. Not least, with respect to the freestyle wrestling, specialized literature underlines that knee joint, hands, wrist, the lumbar and cervical spine, and the area of the trunk are the most common locations for injuries; each of the experienced wrestlers suffered contusions, 55.81% damage to tendons and of articular structures (Brzezińska et al., 2022).

When talking about Mixed-Martial-Arts (MMA) “the injury rate was 9.9 injuries per 100 athletic-exposures (AE)” - for upper limb injuries (Fares et al., 2022), while “head injury rate constituted 35 injuries per 100 athletic-exposures (AE)” - Fares et al. (2021). Among all martial arts and combat sports the most frequent injuries have been damages of knee ligaments (16%) and broken bones (21%) (Cynarski and Kudłacz, 2008). In this context, is very important for specialists to combine psychological and physiological techniques to find preventive actions. In Evans and Brewer (2022) argued that “areas of science in which policy and practice lag behind research evidence are known as *valleys of death* […] and developments in the evidentiary base are required to reach and cross the valley of death to advance the application of psychology to sport injury prevention and rehabilitation over the next 10 years.”

On the severity of injuries in sport, Maffulli and Caine (2005) emphasized a comparison with respect to the existing data in the literature (11 selected studies were investigated and cited), taking into account the time loss from competition, fractures and hospitalization, in: (1) mild (maximum 3 days missed), moderate (4–14 days missed), sever injuries (long-term sequela expected); (2) mild (maximum 1 week time loss), moderate (1–3 weeks time loss), sever injuries (more than 3 weeks time loss); (3) minor (2–7 days time loss), moderate (8–24 days time loss), severe (more than 25 days time loss); (4) minor (maximum 7 days missed), moderate (8–27 days missed), severe (minimum 28 days missed). Also, as Piedade et al. (2021) asserted (talking about martial artists), “in mild injuries the wrestler returns to training and competition according to his symptoms, typically in 2–3 weeks,” while Brzezińska et al. (2022) mentioned that in freestyle wrestling, unfortunately, about 30% of the injuries required surgical treatment. After investigating the existing data in the literature we can conclude that after 2–3 weeks missed/ time loss we are talking about (at least) a moderate injury.

According to The Global Psychological Model of Sports Injury (see Souter et al., 2018), anxiety, stress, depression enhance the risk of injury. Also, Williams and Andersen (1998), in their model of injury and stress, are discussing about personality traits (including anxiety) which can increase distress and the chance of injury in athletes. An A trait-person (person with trait anxiety) is perceiving a broad range of circumstances as being threatening, “and the individual reaction to life events is typically disproportionate to the actual fear properties of the stimulus” (LeUnes, 2008). Considering risk-taking behavior (R-TB), literature highlights instrumental and stimulating risk (Zaleśkiewicz, 2001): instrumental risk is characterized by greater focus on possible losses, the person is reflexive, cognitive processes dominate, as well as reason in decision-making, while stimulating risk is characterized by impulsive, unconscious decision-making and emotional processes dominate. Therefore, R-TB can have two motivations: experiencing pleasurable physiological arousal (stimulating risk) - regardless of the result, or achieving an important goal (instrumental risk). Investigating martial arts athletes, in 2021, Makarowski et al. (2021) emphasized that athletes from Spain registered the lowest results considering stimulating risk, while Romanian martial arts athletes reported the highest values. When talking about the instrumental risk, Russian athletes achieved the lowest values, while Spanish athletes reported the highest scores.

Specialized literature mentions that a high level of anxiety and low coping resources increase the risk of injury in athletes, especially in competition (González-Reyes et al., 2017; Şenışık and Köyağasıoğlu, 2021). Wiese-Bjornstal theory presents the biopsychosocial sport injury risk profile (see Wiese-Bjornstal et al., 1998; Frykholm, 2022) - four categories are taken into account: biological, psychological, physical, and sociological, each holding various levels of risk behavior, risk exposure, and vulnerability to injury.

It was found that anger and fatigue (Galambos et al., 2005), type A athletes - impatient, more aggressive (Nigorikawa et al., 2003), or perfectionistic concerns/ athletes’ perfectionism (but maladaptive perfectionism) - Madigan et al. (2019) are related to an increase likelihood of being injured. Also, meta-analyzes show that neuroticism and extraversion are related to dangerous behaviors, accidents, and injuries (Beus et al., 2015).

In an attempt to identify the athletes’ psychological profile considering the vulnerability to injuries, Andreu et al. (2014) discuss about lower levels of success-oriented motivation and hardiness, and higher levels of motivation oriented to avoid failure and competitive anxiety. It seems that low values on Negative Energy Control and Emotional Stability (Berengüí-Gil et al., 2013), and high scores for Impulsive Sensation Seeking and considering the General Anxiety Disorder Scale (GAD-7) (Madzar et al., 2017) are increasing the likelihood of being injured in athletes. Not least, Berengüí-Gil and Puga (2015) are talking about competitive trait anxiety (high scores) and self-confidence (low values), as predictors of the number of injuries in athletes (in the total sample: athletics - 33.9%, cycling - 12.2%, freestyle wrestling - 21.7%, canoeing - 16.5% and taekwondo - 15.7%). However, in the above prospective study (over a period of 10 months), the number of lesions were counted and correlated to the investigated variables, without providing clear information considering the injury severity. Also, trait anxiety was less examined separately, as in the present research: anxiety of physical danger and anxiety in new, unusual situations, so it’s still worth investigating this topic. We mention a lack of attention to the risk-taking behavior (stimulating and instrumental risk), and A-trait levels (we refer to anxiety in new or ambiguous situations and anxiety in physically dangerous conditions) among martial arts athletes, especially in relation with injury severity.

### 1.1. The current study

The aim of the research is to investigate the level of trait anxiety (in physically dangerous conditions and in new, unusual situations) and the level of risk-taking behavior – R-TB (stimulating and instrumental risk) in competitive martial arts athletes, according to the practiced sports discipline. In the current study we refer to grappling combat sports, striking combat sports, and, also, to striking and grappling sports (Mixed Martial Arts, which combines techniques from both fighting styles), as in previously studies (Predoiu et al., 2022a). At the same time, we wanted to capture the differences between athletes who have suffered at least medium severity injuries and athletes who have not suffered such sports injuries up to the time of testing (considering A-trait and R-TB) and to verify to what extent A-trait and R-TB predict injury severity in martial artists.

### 1.2. Objectives

- Knowing the A-trait and R-TB level in martial arts athletes from striking combat sports, grappling combat sports, respectively striking and grappling (Mixed Martial Artists);
- Identifying the links between injury severity, athletes’ anxiety in new conditions, in physically dangerous circumstances and martial arts’ level of instrumental and stimulating risk;
- Establishing the differences between martial arts athletes, in terms of A-trait and R-TB, taking into account injury severity;
- Identifying predictors of injury severity in the case of martial arts athletes.

*H1*: There are significant differences between martial arts athletes according to the specifics of the sports disciplines (striking combat sports, grappling combat sports, respectively striking and grappling - MMA), in terms of A-trait and risk-taking behavior.

*H2*: There are significant correlations between athletes’ injury severity and martial arts’ level of A-trait and R-TB.

*H3*: Investigation of martial arts athletes who have suffered mild, moderate and/or severe injuries and athletes who have suffered only minor/mild injuries reveals significant differences between the two groups in terms of A-trait and R-TB.

*H4*: The results for R-TB represent a better predictor of injury severity among martial arts athletes than the results obtained for A-trait.

## 2. Materials and methods

### 2.1. Participants

One hundred and fifty-four Romanian martial arts athletes, affiliated to various sports clubs in Romania, participated in the study, 132 male and 22 female, aged 20–32 years (M_age_ = 24.6, SD = 4.12). The athletes had a minimum of 4 years of competitive experience and have been practicing martial arts for an average of 8.39 years, SD = 3.10 (in the entire sample). Inclusion criteria: minimum 4 years of competitive experience, minimum 20 years old (seniors), and minimum 12 matches/ official fights per year. Also, athletes investigated in the current study should not have suffered severe injuries before the investigated period (January 2018–December 2021).

We mention that 188 eligible martial arts athletes (in terms of age and competitive experience) were tested in the initial stages of the study, but 154 remained for future analysis because: seven athletes could not declare the number of official matches/fights (per year) in the last 4 years, because they did not have/no longer have the competition planning and results obtained, nor could they check online (or with the coach) the number of matches held in certain competitions, and eight athletes reported less than 12 official matches/ fights per year (consistently). Also, 19 martial arts athletes reported that they suffered at least one severe injury prior the investigated period (January 2018–December 2021). The reasoning behind the decision to remove from the study the 19 athletes (mentioned above) is linked with the existing challenges, for athletes, when returning in competitions from serious injuries, including anxiety related to injury recovery, anxiety associated with return to play (see, for example, Ogu and Adegbesan, 2013; Bennett and Lindsay, 2016) and fear of reinjury (Cassidy, 2006). By removing these martial arts athletes from the research, we wanted to provide a relatively similar level in terms of the severity of injuries suffered by athletes, at time t_0_ (knowing, also, that psychological and physical readiness to return to sport after sports injuries are not synonymous - Podlog and Eklund, 2006).

The snowball sampling technique was used in the current study due to the specifics of the investigated athletes - part of hard-to-reach population: senior martial arts athletes, having minimum 4 years of competitive experience, having only mild injuries, or minimum one injury of at least medium severity, minimum 12 official matches/ fights per year, and who have not suffered severe injuries before the beginning of the investigation period.

The distribution of the participants is captured in Table 1.

**Table 1:** Descriptive statistics of surveyed martial arts athletes.

Variable	Value
Gender *N* (%)	
Male	132 (85.7%)
Female	22 (14.3%)
Age, M (SD)	24.6 (4.12)
Sports disciplines *N* (%)	
Striking sports disciplines (boxing, karate, kickboxing, taekwondo)	64 (41.5%)–54 male (M) and 10 female athletes (F)
Grappling sports disciplines (judo and jiu-jitsu–BJJ)	39 (25.3%)–31 M and 8 F
Striking and Grappling (Mixed martial arts–MMA)	51 (33.1%)–47 M and 4\u00B0F
Injuries *N* (%)	
Striking sports disciplines	
Minimum one injury of at least medium severity	30 M and 2 F (50%)
	- 14 athletes had only 1 or 2 moderate injuries: grade 2 sprain, tendon injury, dislocation
	- 12 athletes suffered only one severe injury: grade 3 sprain or fracture (e.g., forehead fracture, broken cheekbone/upper jaw, skull fracture, fracture of the shin bone/tibia, aroundh the knee or in the foot)
	- 6 athletes suffered one severe and also 1 or 2 moderate injuries
Only mild injuries	24 M and 8 F (50%) - grade 1 sprain, contusion, concussion, laceration
Grappling sports disciplines	
Minimum one injury of at least medium severity	18 M and 5\u00B0F (59%)
	- 11 athletes had only 1 or 2 moderate injuries: grade 2 sprain, tendon injury, dislocation
	- 8 athletes suffered only one severe injury: grade 3 sprain or fracture (e.g., at the wrist, elbow, shoulder, ankle, around the knee)
	- 4 athletes suffered one severe and also 1 or 2 moderate injuries
Only mild injuries	13 M and 3\u00B0F (41%) - grade 1 sprain, contusion, concussion, laceration
Striking and grappling sports disciplines (MMA)	
Minimum one injury of at least medium severity	29 M and 3\u00B0F (62.7%)
	- 15 athletes had only 1 or 2 moderate injuries: grade 2 sprain, tendon injury, dislocation
	- 10 athletes suffered only one severe injury: grade 3 sprain or fractures (mentioned above)
	- 7 athletes suffered one severe and also 1 or 2 moderate injuries
Only mild injuries	18 M and 1 F (37.3%) - grade 1 sprain, contusion, concussion, laceration
Sports performances *N* (%)	
Striking sports disciplines	
International/national level	20 (31.3%)
Regional/local level	44 (68.7%)
Grappling sports disciplines	
International/national level	15 (38.5%)
Regional/local level	24 (61.5%)
Striking and grappling sports disciplines (MMA)	
International/national level	19 (37.3%)
Regional/local level	32 (62.7%)

It is worth mentioning that the highest risk for time loss (after sports injury, in different sports branches) was registered between 20 and 24 years (Kujala et al., 1995).

In the case of martial artists having minimum one injury of at least moderate severity in the last 4 years, these athletes suffered, also, mild injuries in competitions (but unlike the group with *Only minor injuries*, the differences are clear–see Table 1). When talking about martial arts athletes with only minor/mild injuries, the number of these injuries was not of interest for the current study, being relatively common in competitions and training.

Athletes having international or national level performances were classified as elite/experts (according to the athletes’ highest standard of performance–Swann et al., 2015), while a second group obtained regional or local level results. In both groups (according to the standard of performance) athletes suffered all kinds of injuries–mild, moderate and severe (described in Table 1).

### 2.2. Measures

For assessing trait anxiety (A-trait), the Romanian adaptation of the Endler Multidimensional Anxiety Scales (EMAS) was used (Miclea S. et al., 2009), more exactly, anxiety in new or ambiguous conditions and anxiety in physically dangerous circumstances. EMAS (created by Endler et al., 1991) is a “factorial approach to anxiety assessment,” anxiety being seen as a “multiplicative function of the person–situation interaction” (LeUnes, 2008). There are 15 items in each scale - the items are the same, the martial arts athletes being asked to imagine themselves in the described circumstance. Specifically, for anxiety in physically dangerous situations the standard tutorial was - “We are interested in your reactions to the situations that involve dealing with physically dangerous or potentially harmful things, objects or events,” while for anxiety in new, unusual conditions the tutorial was - “We are interested in your reactions to new, unfamiliar situations and when you are not sure what to expect.” These situations (physically dangerous and new, unusual) are specific, especially, in sports with active and creative opponents, where a greater adaptability of the athlete is needed, as in martial arts, where decisions can be made in a split second.

EMAS was calibrated for the Romanian population by the Cognitrom Company, being part of the CAS^++^ platform (Miclea M. et al., 2009). The interpretation of the results (T scores, automatically generated by the computerized platform, eliminating gender and age differences) is: 45–55 = average results; under 40 = low scores; over 60 = high values; 40–45 = slightly below average results; 55–60 = slightly above average results. Participants responded on a 5-point Likert scale, where 1 - “Not at all,” and 5 denotes “Very much.” Examples of items: “I feel worried,” “I feel comfortable,” “I trust myself,” “I am agitated,” etc. The reliability of the two scales in the present study was verified using the McDonald’s omega coefficient (ω). Considering anxiety in new, ambiguous situations, ω was 0.72, and for anxiety in physically dangerous conditions ω was 0.74 (a satisfactory internal consistency).

In order to assess risk-taking behavior, the Romanian adaptation of the Makarowski’s Stimulating and Instrumental Risk Questionnaire was used (Makarowski et al., 2021). The instrument has 8 items, each scale: stimulating risk and instrumental risk having four items (allowing a rapid assessment of risk levels in athletes). There are five ways of answering, from “a” which denotes True (5 points), to “e” which denotes Untrue (1 point). There are no reverse-scored items. The internal consistency of the two factors was calculated for the current research (using McDonald’s omega coefficient). The values were: ω = 0.79 for stimulating risk, and ω = 0.76 in the case of the instrumental risk.

With respect to the stimulating risk - martial arts athletes are searching for highly stimulating situations, that bring pleasure, being focused on sensation seeking. Athletes do not think about the result/ outcome, it is the actions performed and the pleasure felt that count. Stimulating risk involves a state of excitement and an increase level of physiological arousal. In contrast, instrumental risk implies rational thinking - athletes think about the outcome of the technical executions they perform, choosing risk only when they consider there is a chance of winning (see, also, Predoiu et al., 2022b).

The Desirability Scale of the Zuckerman-Kuhlman Personality Questionnaire (part, also, of the computerized platform CAS^++^, calibrated on the Romanian population - Miclea M. et al., 2009) was used, in order to minimize the likelihood of inadequate responses from athletes (no martial art athlete was removed from the research following the answers to this scale).

An injury report form was created starting from previous studies (e.g., Willick et al., 2013), gathering the following information through 10 questions (close- and open-ended questions were used): (1) gender; (2) age; (3) type of sport; (4) competitive experience (in years); (5) number of official matches/ fights per year - in competitions (from January 2018 to December 2021); (6) highest sports performance; (7) at least one severe injury existing before the investigated period - prior to January 2018 (answer options Yes/ No); (8) injury severity (type of injuries) (a) mild injuries (e.g., grade 1 sprain, contusion, concussion, lacetarion, or something else–a free space was available, also, for athletes to complete, following the listed/available variants) during training or competition, each year (Yes/No), (b) moderate injuries (minim 2–3 weeks missed), e.g., grade 2 sprain, tendon injury, dislocation or something else (a free space was available, also, for athletes to complete, following the mentioned/available variants), (c) severe injuries (months missed/ time loss), e.g., grade 3 sprain or fracture (a free space was available, also, for athletes to complete, following the listed/available variants); (9) place where moderate or severe injury occurred (a) only in competitions/official matches, (b) only during training, (c) in competition or training. A tenth question (close-ended) concerned whether athletes carried out psychology-based counseling programs after injuries, regardless of severity (Yes/ No - 17.5% of martial arts athletes gave a positive answer). At question 8 (a), all martial artists declared that they suffered mild injuries during trainings or competitions (each year), and at question (9) athletes reported that moderate or severe injuries were suffered only in official fights/ matches (in competitions). In this context it is worth mentioning Thomas and Thomas’ systematic review of injuries in martial arts (2018), in which researchers asserted that “there are no studies of training injuries of professionals […] or long-term follow-up of musculoskeletal injuries or neurological damage.”

### 2.3. Research design

The investigation is based on *ex post facto* design - the analysis started (the online surveys were applied) after the fact has occurred, martial art athletes already suffered certain injuries (between January 2018–December 2021) and obtained various sports results in competitions, without interference from the researchers (is a retrospective research).

### 2.4. Procedure

The questionnaires for assessing A-trait, risk-taking behavior, and the injury report form (including socio-demographic information, and data regarding injury severity and the highest sports performance achieved at the time of the survey) were applied *via* Google forms (Google LLC, Mountain View, CA, United States), between March 2022–September 2022. The research was conducted in Romania. We mention that because of the COVID-19 pandemic, for approximately 1 year (starting from March 2020, when the World Health Organization classified the coronavirus disease 2019 as a pandemic), the sports activity (in sports clubs) was constantly interrupted or stopped (the vaccination campaign began, in Romania, on December 27, 2020). During the pandemic “martial arts competitions were organized in Romania (and televised), but without spectators. Only athletes and coaches had access in the competition hall, and they were previously tested against COVID-19” (Predoiu et al., 2022c). In this period, the martial arts athletes from the current research trained exclusively at home (during the lockdown period), and when the conditions relaxed, they practiced outdoors (in parks, on sports fields), respecting the measures of social distancing. Each athlete had minimum 12 official matches/ fights per year (including during the first year of the pandemic), the maximum number of fights (in 1 year) being 25. More exactly, 68.84% of athletes had between 12 and 18 fights per year (in official competitions, M = 15.25, SD = 1.71), while 31.16% - between 19 and 25 matches per year (M = 20.83, SD = 1.80). In the case of martial arts athletes who suffered at least one severe injury - this is the case for 47 of the participants in the current study (30.5%), being out from competitions for several months following the severe injury, matches were counted (minimum 12 matches/fights per year, as inclusion criteria) during 1 year starting from their first competition after recovery. Moderate and severe injuries occurred to athletes in competitions and training in the last 4 years (between January 2018–December 2021) were counted. It is worth mentioning that (in the present study) no athlete reported moderate or severe injuries during trainings (but only mild injuries). Sixteen martial arts athletes had the same moderate injury twice (a recurrent injury), being counted once, the same as in Li et al. (2015) study (see Table 1 for the distribution of athletes according to self-reported injuries).

### 2.5. Statistical analysis

IBM SPSS Statistics Version 27.0 (Armonk, NY, IBM Corp) was used for the statistical analysis. In the case of the single-factor multivariate analysis of variance, taking into account the Levene’s test results (equality of variance–*p* > 0.05), Scheffe post-hoc test was interpreted (Popa, 2010). Using Pearson correlation (r) for parametric tests, relationships between variables were highlighted: strong = 0.51(−0.51) - 0.75(−0.75); weak and moderate = 0.26(−0.26) - 0.50(−0.50); very strong and perfect = 0.76(−0.76) - 1.00(−1.00); null and very weak = 0.00–0.25(−0.25) (Weinberg and Abramowitz, 2002). Considering the effect size for correlation (the coefficient of determination) *r*^2^ = 0.25 - a large effect, *r*^2^ = 0.09 - a moderate effect, *r*^2^ = 0.01 - a small effect (Cronk, 2020). *t*-Test for Independent Samples was also used. Investigated variables were normally distributed, with Skewness coefficients (in absolute value) being less than 1 (Morgan et al., 2004). Finally, analysis of the results involved using binomial logistic regressions, effect size (Nagelkerke *R*^2^) being interpreted as follows: 0.2 small effect size, 0.15 medium, 0.35 large effect size (Cohen, 1992).

## 3. Results

With respect to the preliminary data analysis (stem-and-leaf) no outliers were registered. Also, due to the online survey/submission (all items had to be rated), there were no missing data.

First, we were interested to emphasize if there is a significant association between the level of athletes’ performance (1–international/national, 2–regional/local) and injury severity (1–only mild injuries, 2–one or two moderate injuries, 3–only one severe injury, and 4–athletes suffered one severe injury and one or two moderate injuries), separately, for Striking, Grappling, respectively Striking and Grappling disciplines. The degree of association–Gamma coefficient, between the variables (sports performance and injury severity) was insignificant: Gamma =.

−0.180, *p* = 0.389 (Striking); Gamma = 0.247, *p* = 0.278 (Grappling); Gamma = 0.199, *p* = 0.323 (Striking and Grappling). Therefore, no significant associations were found between the level of sports performances and injury severity in martial arts athletes (analyzed separately, according to the sports disciplines practiced).

The results obtained by athletes for the dependent variables analyzed (at descriptive level), according to the sport discipline practiced are presented in Table 2 (in the case of trait anxiety, results are expressed in T-scores, see *Measures* section for T-scores interpretation).

**Table 2:** Descriptive statistics–A-trait and R-TB, martial arts athletes.

Instrumental risk	Striking combat sports	Mean	17.06
		SD	2.115
	Grappling combat sports	Mean	16.56
		SD	2.479
	Striking and grappling combat sports (MMA)	Mean	16.22
		SD	3.164
Stimulating risk	Striking combat sports	Mean	10.97
		SD	4.276
	Grappling combat sports	Mean	10.82
		SD	3.926
	Striking and grappling combat sports (MMA)	Mean	11.45
		SD	3.936
Anxiety in physically dangerous conditions	Striking combat sports	Mean	38.72
		SD	7.188
	Grappling combat sports	Mean	40.41
		SD	5.665
	Striking and Grappling combat sports (MMA)	Mean	36.78
		SD	6.577
Anxiety in new, unusual situations	Striking combat sports	Mean	47.05
		SD	7.291
	Grappling combat sports	Mean	48.26
		SD	6.695
	Striking and grappling combat sports (MMA)	Mean	46.37
		SD	7.597

Using multivariate analysis of variance (MANOVA) we tested whether there were significant differences between martial arts athletes in the disciplines of Striking, Grappling, Striking and Grappling, respectively, in terms of trait anxiety and risk-taking behavior.

There are weak and very weak positive correlations between the four dependent variables, with the linearity condition being assumed (in the case of MANOVA). The value of the Box M test is insignificant (*p* = 0.181), therefore we refer to the Wilks Lambda test values: Wilks’ Lambda = 0.918, *F*(8, 296) = 1.618, *p* = 0.119. The type I procedure (for group inequality) was selected. Considering the Test of Between-Subjects Effects, the sports discipline practiced significantly influence only the results for anxiety in physically dangerous situations (*F* = 3.361, *p* = 0.037, Partial Eta Squared = 0.043). The homogeneity of variances condition was met - *p* > 0.05 (Levene test), therefore the Scheffe post-hoc test was interpreted (Table 3).

**Table 3:** Post-hoc Scheffe test–single-factor multivariate analysis of variance.

Variables	(I) Sports discipline	(J) Sports discipline	Mean difference (I-J)	*p*
Instrumental risk	Striking combat sports	Grappling	0.50	0.640
		Striking and grappling	0.85	0.224
	Grappling combat sports	Striking	−0.50	0.640
		Striking and grappling	0.35	0.819
	Striking and grappling combat sports (MMA)	Striking	−0.85	0.224
		Grappling	−0.35	0.819
Stimulating risk	Striking combat sports	Grappling	0.15	0.984
		Striking and grappling	−0.48	0.820
	Grappling combat sports	Striking	−0.15	0.984
		Striking and grappling	−0.63	0.768
	Striking and grappling combat sports (MMA)	Striking	0.48	0.820
		Grappling	0.63	0.768
Anxiety in physically dangerous conditions	Striking combat sports	Grappling	−1.69	0.456
		Striking and grappling	1.93	0.302
	Grappling combat sports	Striking	1.69	0.456
		Striking and grappling	3.63	**0.039**
	Striking and grappling combat sports (MMA)	Striking	−1.93	0.302
		Grappling	−3.63	**0.039**
Anxiety in new, unusual situations	Striking combat sports	Grappling	−1.21	0.714
		Striking and grappling	0.67	0.885
	Grappling combat sports	Striking	1.21	0.714
		Striking and grappling	1.88	0.476
	Striking and grappling combat sports (MMA)	Striking	−0.67	0.885
		Grappling	−1.88	0.476

Significant differences between martial arts athletes by sport discipline were found only in the case of anxiety in physically dangerous situations–*p* = 0.039 (Table 3), between the sports disciplines of Grappling (M_GRAPPLING_ = 40.41, SD = 5.66) and Striking and Grappling (MMA) - M_STRIKING and GRAPPLING_ = 36.78, SD = 6.57.

Next, we checked the existing relationships between injury severity, A-trait and R-TB scores in martial artists, separately, according to the sports disciplines. In the case of injury severity: 1–only minor/mild injuries, 2–one or two moderate injuries, 3–only one severe injury, and 4–athletes suffered one severe injury and one or two moderate injuries. Table 4 contains only the significant relationships between variables.

**Table 4:** Correlation (Pearson r) between injury severity, A-trait and R-TB scores.

		IS	A	B	C	D
Striking combat sports						
D	*r*	0.284	−0.134	0.346	0.643	1.000
	*p*	0.115	0.464	0.053	0.000^***^	.
Grappling combat sports						
D	*r*	0.171	−0.242	0.128	0.761	1.000
	*p*	0.436	0.267	0.561	0.000^***^	.
Striking and Grappling (MMA)						
C	*r*	0.408	−0.181	0.441	1.000	0.416
	*p*	0.020^*^	0.321	0.012^*^	.	0.018^*^

IS, Injury severity; A, Instrumental risk; B, Stimulating risk; C, Anxiety in physically dangerous conditions; D, Anxiety in new, unusual situations.

*p < 0.05; ***p < 0.001.

Table 4 emphasizes no significant correlations between injury severity and results for A-trait and R-TB (*p* > 0.05) in the case of both striking and grappling combat sports. However, a significant positive correlation was observed (*r* = 0.643, *p* < 0.001, the coefficient of determination/ effect size *r*^2^ = 0.41–in striking combat sports; *r* = 0.761, *p* < 0.001, *r*^2^ = 0.58–in grappling combat sports) between anxiety in new situations and anxiety in physically dangerous circumstances. In the case of Mixed Martial Arts athletes a significant relationship between injury severity and anxiety in physically dangerous situations was found (*r* = 0.408, *p* = 0.020, *r*^2^ = 0.16). A higher score for this facet of anxiety (at group level athletes scored below average, according to the standard/norms) is associated with more severe injuries. We can say that 16% of the total variation is shared/common between the two variables, the rest being due to other influences (the relation between injury severity and anxiety in physically dangerous conditions is moderate to strong; 95% confidence interval: lower limit = 0.15 and upper limit = 0.614). Also, the results in Table 4 emphasize a significant positive correlation between anxiety in physically dangerous circumstances and: stimulating risk (*r* = 0.441, *p* = 0.012, *r*^2^ = 0.19), respectively anxiety in new situations (*r* = 0.416, *p* = 0.018, *r^2^* = 0.17).

To verify whether there are significant differences in terms of trait anxiety and risk-taking behavior between martial arts athletes who have suffered mild, moderate and/or severe injuries (*n* = 87) and athletes who have suffered only minor/mild injuries - *n* = 67, t-Test for Independent Samples was used (dependent variables were normally distributed, the skewness values being less than 1). The results for descriptive statistics (in the case of A-trait and R-TB, according to injury severity) are highlighted in Table 5.

**Table 5:** Descriptive statistics–martial arts athletes results for A-trait and R-TB according to injury severity (regardless of sports disciplines).

Dependent variables		Mean	SD	SE
Instrumental risk	Mild, moderate and/or severe injuries	16.44	2.92	0.314
	Only minor/mild injuries	17.22	1.81	0.222
Stimulating risk	Mild, moderate and/or severe injuries	11.41	4.06	0.436
	Only minor/mild injuries	10.67	4.04	0.494
Anxiety in physically dangerous conditions	Mild, moderate and/or severe injuries	38.83	6.60	0.708
	Only minor/mild injuries	38.09	6.92	0.846
Anxiety in new, unusual situations	Mild, moderate and/or severe injuries	47.86	6.76	0.725
	Only minor/mild injuries	45.51	7.00	0.855

The results in Table 6 underline that the average value for instrumental risk is significantly higher [*t* = −2.05, *p* = 0.042] in martial arts athletes who have suffered only mild injuries (obtained a high score, according to the norms - see Makarowski et al., 2021) compared to athletes who have suffered mild, moderate and/or severe injuries (registered a lower score). When talking about anxiety in new, unusual situations, we emphasized, also, a significant difference [*t* = 2.11, *p* = 0.037] between athletes who have suffered only mild injuries and those who experienced more severe injuries (the results, for both groups, ranged from 45 to 55 T-scores, meaning average results). Hedge’s g (the effect size index) = 0.31 (instrumental risk), respectively *g* = 0.34 (anxiety in new conditions), a moderate to weak difference (between the two investigated groups) being emphasized.

**Table 6:** Inferential statistics–independent sample *t* test.

Variables		Levene’s test						
		F	*p*	*t*	df	*p*	Confidence interval	
							Lower	Upper
A	Unequal variances	9.49	0.002	−2.05	145.9	0.042	−1.546	−0.028
B	Equal variances	0.012	0.914	1.125	152	0.262	−0.561	2.045
C	Equal variances	0.558	0.456	0.673	152	0.502	−1.427	2.904
D	Equal variances	0.100	0.753	2.110	152	0.037	0.149	4.560

A, Instrumental risk; B, Stimulating risk; C, Anxiety in physically dangerous conditions; D, Anxiety in new, unusual situations.

In the next phase, knowing that instrumental risk and anxiety in new, unusual situations are important psychological variables considering injury severity among martial arts athletes, we verified to what extent the two psychological dimensions predict injury severity. To achieve this goal two separate logistic regressions (binomial) were performed.

The models are statistically significant (Table 7, *p* < 0.05, Omnibus test - Model). In the case of the Hosmer and Lemeshow goodness of fit test, *p* = 0.698 (for instrumental risk) and *p* = 0.141 (for anxiety in new situations) emphasizing that the models are not a poor fit. The logistic regression models were statistically significant: instrumental risk - *χ*^2^(1) = 4.44, *p* = 0.035; anxiety in new conditions - *χ*^2^(1) = 4.76, *p* = 0.029. In the case of martial arts athletes, the results for A-trait in new or unusual situations represent a slightly better predictor of injury severity than the values for instrumental risk - one can observe that the differences between the two models are almost non-existent, both psychological factors having the capacity to predict injury severity in martial arts athletes. The models correctly classified 56.7% (instrumental risk), respectively 58.4% (anxiety in new, unusual circumstances) of cases. The contribution of the two psychological phenomena in predicting injury severity is important, representing valuable resources for athletes, sports psychologists, physiotherapists, present and future martial arts coaches. Nagelkerke *R*^2^ (effect size index) shows a moderate to weak relation between both psychological dimensions and injury severity. We can assert that a moderate or slightly below average level of anxiety in new situations and a higher level of instrumental risk are associated with a decreased likelihood of severe injuries in martial arts athletes.

**Table 7:** Results of the binomial logistic regressions analysis.

	Instrumental risk	Anxiety in new situations
Omnibus test–model (Chi-square, value of *p*)	4.44 (0.035)	4.76 (0.029)
Hosmer and Lemeshow test (Chi-square, value of *p*)	5.54 (0.698)	8.29 (0.141)
Nagelkerke *R*^2^	0.048	0.051
Overall percentage (Predicted–Percentage correct)	56.7	58.4
Wald test	4.266	4.365
*B*	−0.051	0.149
SE	0.025	0.071
*Odds ratio* values	1.052	0.862
Confidence interval for *Exp(B)*	1.009–1.104	0.750–0.991

## 4. Discussion

In sports field, severe injuries may predispose athletes to mental health issues such as depression, anxiety, distress, to adverse health behaviors and lower self-esteem (Gulliver et al., 2015). Martial arts and combat sports suppose a behavioral code which includes moral and ethical values (Makarowski et al., 2021). But the “win-at-all-costs philosophy” in competition, which manifests, especially, among youth athletes, can generate violence and hostile aggression (Urzeală and Teodorescu, 2018), leading to sports injuries. Maybe the most important question which the coach, athlete, parents etc. ask is “When will I/ she /he be able to play again?.” (Gomez-Piqueras et al., 2018). Specialized literature asserted that in combat sports (judo) mean absence from competition and training after injuries sustained during the Summer Olympic Games (London) was comprised between 1 and 7 days (Engebretsen et al., 2013), while in the case of lower level competitions mean absence ranged from 21 to 29 days (Green et al., 2007). However, there are researchers who found the opposite - higher injury rate as sports performance increases (Bauer and Steiner, 2009). The current study underlined that no significant relationships exists between the level of sports performances and injury severity in martial arts athletes (investigated separately - striking combat sports, grappling combat sports and mixed martial artists). It seems that other variables have a more important role (than competition level, in martial artists) with respect to athletes’ injury severity.

In a first phase we highlighted the existence of significant differences between martial arts athletes, taking into account the sports disciplines, for anxiety in physically dangerous situations. Athletes from grappling combat sports (judo and BJJ) registered a slightly below average level of anxiety in physically dangerous conditions, while athletes from striking and grappling combat sports (MMA) obtained a low score. No significant differences between martial arts athletes from striking sports, grappling sports disciplines and Mixed Martial Arts were found, in terms of instrumental and stimulating risk, or considering anxiety in new situations.

Further we verified the existing correlations between injury severity, A-trait and risk-taking behavior (R-TB) in athletes, separately, according to the sports disciplines practiced. Only in the case of striking and grappling combat sports (MMA) a significant relation between injury severity and anxiety in physically dangerous situations was found. A higher score for this facet of anxiety is linked with more severe injuries. Although anxiety in physically dangerous conditions is below average (at group level), Mixed Martial Arts athletes having a lower score (highly below average) are at an advantage, considering the severity of recorded injuries.

Even if no significant correlations between injury severity and results for A-trait and R-TB were found in the case of athletes from striking combat sports (karate, taekwondo, boxing and kick-boxing) and practicing grappling combat sports (judo and jiu-jitsu), the following associations were, however, observed, between: (1) anxiety in new situations and anxiety in physically dangerous conditions (in both groups - striking and grappling, but also in MMA athletes); (2) anxiety in physically dangerous conditions and stimulating risk (in MMA). One can observe that: martial arts athletes with higher scores for anxiety in new, unusual situations registered, also, a higher anxiety in potentially harmful situations (linked with more severe injuries in MMA); athletes from Mixed Martial Arts with higher scores for anxiety in physically dangerous conditions analyze less rationally the situations in competition, instead they are more adrenaline-seeking in competition, take more pleasure in just performing technical executions without thinking about success or failure, engaging more in risky actions. These results are consistent with studies noting that more anxious individuals exhibit higher stimulating risk, in other words, they act more according to the *all-or-nothing* principle (Makarowski et al., 2016). The outcome in MMA athletes - more moderate and/or severe injuries reported (we must not forget the above mentioned relation between injury severity and anxiety in physically dangerous situations in Mixed Martial Arts athletes).

In the next phase, the existing differences considering trait anxiety and risk-taking behavior between martial arts athletes who have suffered mild, moderate and/or severe injuries and athletes who have suffered only minor/mild injuries were examined (regardless of combat sports disciplines). The results emphasized that the values for instrumental risk are significantly higher in athletes who suffered only mild injuries, compared to those who have experienced mild, moderate and/or severe injuries. The scores were, instead, lower (in the first group) with respect to anxiety in new, unusual situations (a moderate to weak difference between the results was highlighted). Martial arts athletes who think more about the outcome of the technical executions they perform (use more rational thinking), choosing risk only when they consider there is a chance of winning and searching less for highly stimulating situations, reported less severe injuries. The same aspect was observed (less severe injuries) in athletes having a lower level of anxiety in new, unusual situations.

Not least, we were interested in highlighting to what extent anxiety in new situations and instrumental risk predicts injury severity among martial arts athletes. We can argue that A-trait in new, unusual situations represent a slightly better predictor of injury severity than instrumental risk. A moderate or slightly below average level of anxiety in new circumstances and a higher level of instrumental risk are associated with a decreased likelihood of severe injuries in martial arts athletes. Ivarsson and Johnson (2010) found, also, that “somatic trait anxiety, psychic trait anxiety, stress susceptibility, and trait irritability” represent important psychological dimensions, predicting injuries in athletes (soccer players were examined), while Nippert and Smith (2008) asserted that personality traits and stress generated by various life situations have the potential to influence the occurrence of sports trauma. The predictor variables in the current study represent valuable resources for present and future sports psychologists, coaches, physiotherapists and medical practitioners. We mention a gap in the literature considering the instrumental and stimulating risk level of martial arts athletes and its connection with sports injuries.

Specialists need to be aware of the psychological impact that sports injuries entail (Walker et al., 2007). As Van Niekerk and Lynch (2012) asserted “the high level of anxiety associated with players who suffered shoulder injuries has to be targeted with anxiety management skills as part of a player development and injury management program.” In the case of martial arts athletes, in order to reduce trait anxiety and distress (e. g., from negative life event–an indirect effect on injury frequency, see Ivarsson et al., 2012), for athletes’ personal development, and for injury prevention, various techniques and psychology-based counseling programs could be used. These may involve: stress management strategies, containing visualization, relaxation, emotional relief, self-monitoring of emotional reactions, cognitive restructuring (Perna et al., 2003); analytical relaxation and autogenic training, increasing self-confidence (Gould et al., 2002); *internal* techniques (breathing, meditation), reducing state hostility and aggressive impulses in martial arts athletes (Hernandez and Anderson, 2015); positive self-talk (inner monolog) and a greater involvement in tasks which gives satisfaction to athletes (Lyubomirsky et al., 2005); social support, strengthening the athlete’s coping resources (Junge, 2000). Also, biofeed-back training together with cognitive-behavioral training seems to be a performant strategy to decrease the occurrence of sports’ injuries (Edvardsson et al., 2012), while Vasile (2022) discusses about “an integrative model of intervention against psychological (chronical and traumatic) stress,” involving the following aspects: emotional, cognitive, and behavioral (body). After a sport injury occurs, positively reframing and emotional support helped athletes with serious sport-related injuries to persevere (Podlog and Eklund, 2007; Salim et al., 2016), while verbal emotional disclosure played an important role in the sport injury-related growth (Salim and Wadey, 2018). Returning from sports injuries presents, therefore, numerous challenges, including the anxiety surrounding injury recovery. Bennett and Lindsay (2016) present a detailed intervention program in this situation (considering anxiety related to injury recovery), focusing on “mindfulness and relaxation strategies to reduce imagined pain associated with an old injury and manage anxiety associated with return to play.” Mohammed et al. (2018) highlight, also, the benefits of a Mindfulness Based Stress Reduction intervention to decrease anxiety/stress and increase pain tolerance of injured athletes.

The results of the current research extend previous studies and addressed gaps in literature. Regarding the limitations of the study, one could argue that each combat sport and martial art discipline should be separately investigated. New studies might focus, therefore, on one specific sports branche (e.g., boxing, judo, taekwondo, etc.), taking into account, also, that “individual sport athletes are more likely to report anxiety and depression than team sport athletes” (Pluhar et al., 2019). However, anxiety of injury did not vary significantly when athletes from individual or team sports were examined (Tanyeri, 2019).

Taking into account the research design (a retrospective study using online survey) and that “the frequency of fractures demonstrated in retrospective studies based on institutional documentation (RD) was considerably higher than in retrospective studies utilizing questionnaires (RQ) and in prospective studies “(Pocecco et al., 2013), further RD studies and prospective cohort researches (longitudinal studies that follows martial arts athletes over time, to determine how A-trait and risk-taking behavior affect injury rate in competition and training) are needed. Also, an equal number of fights/ competitions (for each athlete) could be taken into account.

In the present research self-report tools were used, being known the possible recall bias/ memory bias when talking about explicit measures (Predoiu et al., 2022c) and the problem of possible desirable responses. The social desirability scale was used to minimize the risk of athletes giving inappropriate answers, the relatively large number of participants represents a strength of the paper, and it is worth mentioning that questionnaires were used, also, in previously studies which investigated injury severity (e.g., Williams et al., 2020; Roșu, 2022). Moreover, as Rădoi et al. (2019) asserted, “self-report measures [...] are critical tools for identifying patients with persistent post-concussion symptoms and their follow-up.”

A-trait and risk-taking behavior were investigated after the events occurred (*ex post facto*), after martial arts athletes suffered different sports injuries (in the last 4 years) and after various events in athletes’ life, so we cannot assert to what extent (1) injuries suffered, or other stressful events, have generated a higher anxiety in new conditions, or a lower level of instrumental risk, taking into account Williams and Andersen’s theoretical stress-injury model (1998) - a stressful event, which could originate, also, outside of the sport context, could affect athletes’ reactions, generating distractibility, reduced ability to analyze specific situations in competition, increased muscle tension and, as a consequence, could increase injury risk, or (2) A-trait and risk-taking behavior, as personality traits (relatively stable at personality level, which hardly changes - Ciolcă et al., 2019) influenced injury severity. The present study is a retrospective one, based on questionnaires, which emphasized that anxiety in new, unusual conditions and instrumental risk (played the role of the independent variables) are important predictors of injury severity in martial arts athletes.

Not least, another facets of trait anxiety must be investigated, for example, separation anxiety, only training injuries because studies of training injuries of competitive martial arts athletes are missing (Thomas and Thomas, 2018), or other important aspects in relation to sports injuries, such as: competitive system, athletes’ exercise capacity, recovery and nutrition, etc.

## 5. Conclusion

In summary, we emphasize that a moderate or slightly below average level of anxiety in new, unusual conditions and a higher level of instrumental risk are associated with a decreased likelihood of severe injuries in martial arts athletes. Additionally, we highlight that in the case of striking and grappling combat sports (MMA) a higher score for anxiety in physically dangerous situations is linked with more severe injuries. Athletes from Mixed Martial Arts with higher scores for anxiety in physically dangerous conditions analyze less rationally the situations in competition, instead they seek more adrenaline, engaging more in risky actions, while athletes from striking combat sports (boxing, kick-boxing, karate, taekwondo) who are more anxious in new situations are more adrenaline-seeking, taking more pleasure in just performing technical executions regardless of the outcome. Not least, martial arts athletes (in entire sample) who have suffered mild, moderate and/or severe injuries, compared to athletes who suffered only minor/mild injuries, look for more highly stimulating situations in competition, and use less rational thinking when evaluating the outcome of the technical executions they perform.

## Data availability statement

The raw data supporting the conclusions of this article will be made available by the authors, without undue reservation.

## Ethics statement

The studies involving human participants were reviewed and approved by the local ethics committee of the National University of Physical Education and Sport, Bucharest, authorization number assigned is ID:946. Data were treated confidentially and the anonymity of the participants was ensured. The patients/participants provided their written informed consent to participate in this study.

## Author contributions

All authors listed have made a substantial, direct, and intellectual contribution to the work and approved it for publication.

## Conflict of interest

The authors declare that the research was conducted in the absence of any commercial or financial relationships that could be construed as a potential conflict of interest.

## Publisher’s note

All claims expressed in this article are solely those of the authors and do not necessarily represent those of their affiliated organizations, or those of the publisher, the editors and the reviewers. Any product that may be evaluated in this article, or claim that may be made by its manufacturer, is not guaranteed or endorsed by the publisher.
